# Evaluating the impact of policies recommending PrEP to subpopulations of men and transgender women who have sex with men based on demographic and behavioral risk factors

**DOI:** 10.1371/journal.pone.0222183

**Published:** 2019-09-19

**Authors:** Holly Janes, Marshall D. Brown, David V. Glidden, Kenneth H. Mayer, Susan P. Buchbinder, Vanessa M. McMahan, Mauro Schechter, Juan Guanira, Martin Casapia

**Affiliations:** 1 Vaccine and Infectious Disease Division, Fred Hutchinson Cancer Research Center, Seattle, Washington, United States of America; 2 Department of Epidemiology and Biostatistics, University of California School of Medicine, San Francisco, California, United States of America; 3 Division of Infectious Diseases, Beth Israel Deaconess Medical Center, and The Fenway Institute, Fenway Health, Boston, Massachusetts, United States of America; 4 Bridge HIV, San Francisco Department of Public Health, San Francisco, California, United States of America; 5 Department of Medicine, University of Washington, Seattle, Washington, United States of America; 6 Projeto Praça Onze, Universidade Federal do Rio de Janeiro, Rio de Janeiro, Brazil; 7 Asociación Civil Impacta Salud y Educación, Lima, Peru; 8 Asociación Civil Selva Amazónica, Iquitos, Peru; Northwestern University, UNITED STATES

## Abstract

**Introduction:**

Developing guidelines to inform the use of antiretroviral pre-exposure prophylaxis (PrEP) for HIV prevention in resource-limited settings must necessarily be informed by considering the resources and infrastructure needed for PrEP delivery. We describe an approach that identifies subpopulations of cisgender men who have sex with men (MSM) and transgender women (TGW) to prioritize for the rollout of PrEP in resource-limited settings.

**Methods:**

We use data from the iPrEx study, a multi-national phase III study of PrEP for HIV prevention in MSM/TGW, to build statistical models that identify subpopulations at high risk of HIV acquisition without PrEP, and with high expected PrEP benefit. We then evaluate empirically the population impact of policies recommending PrEP to these subpopulations, and contrast these with existing policies.

**Results:**

A policy recommending PrEP to a high risk subpopulation of MSM/TGW reporting condomless receptive anal intercourse over the last 3 months (estimated 3.3% 1-year HIV incidence) yields an estimated 1.95% absolute reduction in 1-year HIV incidence at the population level, and 3.83% reduction over 2 years. Importantly, such a policy requires rolling PrEP out to just 59.7% of MSM/TGW in the iPrEx population. We find that this policy is identical to that which prioritizes MSM/TGW with high expected PrEP benefit. It is estimated to achieve nearly the same reduction in HIV incidence as the PrEP guideline put forth by the US Centers for Disease Control, which relies on the measurement of more behavioral risk factors and which would recommend PrEP to a larger subset of the MSM/TGW population (86% vs. 60%).

**Conclusions:**

These findings may be used to focus future mathematical modelling studies of PrEP in resource-limited settings on prioritizing PrEP for high-risk subpopulations of MSM/TGW. The statistical approach we took could be employed to develop PrEP policies for other at-risk populations and resource-limited settings.

## Introduction

Pre-exposure prophylaxis (PrEP) with tenofovir disoproxil fumarate (TDF)-based oral antiretroviral regimens has been shown to be efficacious for preventing HIV acquisition in cisgender men who have sex with men (MSM), transgender women (TGW), HIV serodiscordant heterosexual couples, and people who inject drugs, with less consistent results among cisgender women [1–7]. Low adherence is likely a major factor explaining the variable efficacy across trial populations [8–10], although biological and behavioral factors may also play a role [11–18].

PrEP delivery requires considerable public health infrastructure to maximize adherence and to screen PrEP users regularly for renal safety, sexually transmitted infections (STIs), and incident HIV infection to prevent PrEP use post-infection; drug resistance is also possible [1–5]. With the licensing of oral co-formulated TDF and emtricitabine (FTC) (FTC-TDF) as PrEP [19], the World Health Organization (WHO) and the US Centers for Disease Control and Prevention (CDC) have disseminated guidelines for the use of PrEP for HIV prevention. In developing such guidelines, the population incidence of HIV, expected PrEP effectiveness, cost of medical care and infrastructure associated with PrEP delivery, and access to PrEP must be considered. Furthermore, in resource-limited settings, policies that prioritize PrEP for select subpopulations warrant consideration.

Inspired by the approaches to policy development and evaluation in other clinical contexts [20–25], we used data from iPrEx, the largest PrEP efficacy trial to date in MSM/TGW, to identify subpopulations of MSM/TGW who could be prioritized for PrEP rollout in resource-limited settings. We relied on a decision-theoretic framework, under which the optimal policy is that which maximizes population net benefit [26–32]. The optimal policy would recommend PrEP to subpopulations with the highest absolute reduction in HIV incidence due to PrEP. We call this a “PrEP-benefit-based policy”. We also considered a “risk-based” PrEP policy, similar in concept to those put forth in the WHO and CDC guidelines [33, 34], which recommends PrEP to individuals at high risk of HIV acquisition without PrEP. A risk-based policy would achieve the same population impact as a PrEP-benefit based policy if the effect of PrEP was a constant reduction in risk of HIV, i.e. if there was no modification of the PrEP effect on the relative risk scale [28, 29, 35, 36]. Under this assumption, the reduction in absolute HIV risk due to PrEP is proportional to risk of HIV without PrEP, and thus high-risk subpopulations have the largest absolute reduction in HIV incidence due to PrEP. This may be the implicit assumption underlying the risk-based policies used in existing guidelines. If, however, demographic or risk behavior characteristics modify the relative risk associated with PrEP, then subgroups at high risk of HIV without PrEP may not be those with the highest benefit from PrEP. We evaluated both risk- and benefit-based PrEP policies to explore this possibility. Specifically, we fit statistical models to the iPrEx data to identify both MSM/TGW subpopulations at high risk of HIV acquisition without PrEP, and subpopulations with high expected PrEP benefit. Next, we used these models to define risk- and PrEP-benefit-based policies, and determine the size of the MSM/TGW subpopulations who would be recommended to take PrEP and the expected HIV incidence under each policy. We compared these data-driven policies- optimized using the iPrEx data- to the existing PrEP guidelines in terms of population impact. Using the iPrEx data, we estimated the population impact of policies empirically- without reliance on modelling assumptions.

Mathematical modelling towards cost-effectiveness analysis has been the primary tool for assessing the population impact of PrEP [37–42], but the modelling has not evaluated potential prioritization based on data-driven statistical models of PrEP benefit or of HIV risk. Math-model-based population impact estimates also rely on many assumptions, such as population distributions of demographic characteristics, risk behaviours, and adherence, and on the associations between these factors and PrEP efficacy. Empirical estimates of the impact of PrEP policies, which do not rely on these assumptions, are lacking.

As for any analyses of randomized trial data, our results based on the iPrEx data pertain directly to the population enrolled in the trial, and additional data are needed to inform on the impact of PrEP policies for other populations. Of particular importance is adherence, since data suggest that adherence in iPrEx was considerably lower than in subsequent open-label and observational studies, and in settings where individuals are being provided an intervention they know to be effective [4, 5, 43–50]. Future research will be needed to determine if the impact estimates based on iPrEx generalize to populations with other distributions of adherence, as well as different demographic and risk behavior characteristics. We elaborate on this in the discussion.

## Materials and methods

### Ethics statement

The iPrEx study [1] was approved by the Committee on Human Research at the University of California, San Francisco, as well as local institutional review boards at each study site: Comité Institucional de Bioética, Asociación Civil Impacta Salud y Educación, Lima, Peru; Universidad San Francisco de Quito, IRB #1, Quito, Ecuador; Fenway Community Health Institutional Review Board, Boston, MA; Comissão de Ética para Análise de Projetos de Pesquisa, CAPPesq Hospital das Clínicas da Faculdade de Medicina da USP, São Paulo, Brazil; Comitê de Ética em Pesquisa, Hospital Universitario Clementino Fraga Filho/Universidade Federal de Rio de Janeiro, Rio de Janeiro, Brazil; Comitê de Ética em Pesquisa do Instituto de Pesquisa Clínica Evandro Chagas, Rio de Janeiro, Brazil; National IRB: Comissão Nacional de Ética em Pesquisa–CONEP, Ministério da Saúde, Brasília, Brazil; University of Cape Town Research Ethics Committee, Cape Town, South Africa; Human Experimentation Committee, Research Institute for Health Sciences, Chiang Mai, Thailand; Ethical Review Committee for Research in Human Subjects, Department of Medical Services, Ministry of Public Health, Nonthaburi, Thailand; Research Ethics Committee, Faculty of Medicine, Chiang Mai University, Chiang Mai, Thailand. Written informed consent was obtained from each participant prior to enrollment in the study.

### The iPrEx study

The iPrEx trial was a phase III study of FTC-TDF for HIV prevention; the results of the primary analysis of safety and efficacy were published by Grant et al. [1] Enrolment began in July 2007; participants were followed until November 2010. The trial is registered at ClinicalTrials.gov and the clinical trial number is NCT00458393. The URL is https://clinicaltrials.gov/ct2/show/NCT00458393.

iPrEx enrolled 2499 HIV-uninfected MSM and TGW who were randomized to placebo or oral FTC-TDF once daily and followed for incident HIV infection. A total of 2442 participants were included in our analysis (10 were HIV-infected at enrolment and 47 did not have a follow-up HIV test). At November 21, 2010, median follow-up was 1.66 years (range: 0.07 to 3.30). The estimated efficacy of PrEP was 44% (p = 0.005). Eighty-three incident infections occurred in the placebo arm, yielding an annual HIV incidence of 4.01% (95% confidence interval [CI]: 2.89–5.08%). Forty-eight infections occurred in the FTC–TDF arm (annual HIV incidence = 1.97%, 95% CI: 1.16–2.86%). Thus, PrEP was estimated to yield a 2.04% absolute reduction in the 1-year rate of HIV infection (95% CI: 0.66%-3.55%, p = 0.003), corresponding to a number needed to treat (NNT) of 49 participants treated per HIV infection event prevented. The efficacy of PrEP on the relative risk scale was estimated to be 44% [1]. Sub-optimal adherence among trial participants likely explains the modest efficacy [51].

Interviewer-administered or computer-assisted questionnaires were used to collect demographic and behavioral risk data on all participants at trial screening. Sexual risk-taking behaviours pertain to the prior 3 months; and exchange of sex for money, drugs, or services and self-reported STIs cover the prior 6 months.

### Risk and PrEP benefit modelling

We considered participants’ age, gender identity, and self-reported sexual risk behaviours at baseline to predict HIV risk and PrEP benefit. Importantly, while demographic and risk behaviour characteristics are frequently collected in clinical practice, measures of adherence are not available before PrEP is actually provided. Accordingly, measures of adherence to FTC-TDF were not included in our models of HIV risk or PrEP benefit. Categorical demographic/risk behaviour variables with less than two HIV cases per level were excluded to improve model stability.

We used Cox proportional hazards logic regression models [52, 53] to select the individual variables or combinations of variables that best predict risk of HIV infection without PrEP, given data for participants on the placebo arm. We paired the fitted Cox model with a Nelson-Aalen baseline hazard estimate [54] to estimate the cumulative HIV infection rate without PrEP, denoted by Risk_0_(*X*), where *X* is a vector of baseline demographic and behavioral variables. We used the same approach to predict HIV risk under PrEP using data from the FTC-TDF arm, denoted by Risk_1_(*X*). We calculated PrEP benefit as the difference in HIV risk without vs. with PrEP, Δ(*X*) = Risk_0_(*X*) − Risk_1_(*X*). Cross-validation was used to select the tuning parameters for the logic regression models. To assess model stability, models were re-fit in 500 bootstrap samples. Further details are summarized in supplementary materials, S1 Methods.

### PrEP policies

We first considered the policy that maximizes net benefit, which recommends PrEP to individuals with a high expected benefit from PrEP as measured by Δ(*X*) = Risk_0_(*X*)–Risk_1_(*X*) [26–32]. The optimal threshold of PrEP benefit above which PrEP is recommended corresponds to the inverse of the *threshold number needed to treat*–the maximum number of individuals one is willing to treat to prevent one HIV infection [55–57]. We focused primarily on a threshold of 1.2%, after considering the null hypothesis used to design iPrEx and other PrEP efficacy trials [1, 2, 6]. The null hypothesis codifies the design assumptions about the NNT to make PrEP clinically useful. Specifically, a null of 30% PrEP efficacy and 4.0% 1-year HIV incidence in the placebo group implies that 1.2% is the smallest absolute reduction in 1-year HIV incidence due to PrEP that would justify PrEP for HIV prevention. The 1.2% threshold corresponds to an NNT of 83. We also considered the effect of different PrEP benefit thresholds.

The second type of PrEP policy we considered was motivated by current WHO guidelines, which suggest considering PrEP for sub-populations with 3 or more HIV infections per 100 person-years at risk [58]. When there is no modification of the PrEP effect on the relative risk scale, an individual’s level of PrEP benefit, Δ(*X*) = Risk_0_(*X*)–Risk_1_(*X*), is proportional to their risk of HIV without PrEP, Risk_0_(*X*)–and therefore high-risk individuals have the largest PrEP benefit. However, if this assumption does not hold, due to effect modification on the relative risk scale, a risk-based policy may have less population impact than a PrEP-benefit based policy. We evaluated a risk-based policy, which recommends PrEP to individuals with a 1-year HIV risk of 3% or more, consistent with the WHO guidelines. We compared this policy to the US CDC PrEP guidelines for MSM that recommends PrEP on the basis of 7 demographic and risk factors including number of male partners and condomless intercourse [33] (Table 1), and to the PrEP-benefit based policy defined above. The guidelines are based on a clinical screening index that was developed using data from VAXGEN 004, an HIV vaccine trial among MSM in the US [59, 60], and validated using data from Project Explore, an HIV behavioral intervention trial among US MSM [61].

**Table 1:** US CDC recommended indications for use of PrEP among MSM [33] and methods used to determine associated PrEP recommendations for iPrEx trial participants, given baseline demographic and risk behaviour data^a^.

CDC criterion	Criterion met for iPrEx participants?
Adult man	Yes, considered satisfied for all participants^b^
Without acute or established HIV infection	Yes, all participants
Any male sex partners in last 6 months	Yes, all participants
Not in a monogamous partnership with a recently-tested, HIV-negative man	Yes if > 1 male partner *OR* HIV-positive partner in last 3 months
*AND* at least one of the following:	
Any anal sex without a condom in last 6 months	Yes if condomless intercourse in last 3 months
Any STI diagnosed or reported in last 6 months	Yes if STI reported in last 6 months *OR* seropositive for syphilis
Is in an ongoing sexual relationship with an HIV-positive male partner	Yes if HIV-positive partner in last 3 months

MSM, men who have sex with men; PrEP, pre-exposure prophylaxis; STI, sexually transmitted infection.

^a^At screening, iPrEx participants were asked about sexual risk-taking behaviours over the prior 3 months. Questions about exchange of sex for money, drugs, or services and self-reported sexually transmitted infections (STIs) covered the last 6 months.

^b^CDC guidelines do not specify special considerations for TGW. Therefore, we applied the same criteria to both MSM and TGW in iPrEx.

### Evaluating population impact

We used existing methods to assess the population impact of PrEP policies [62–65]. Each policy was first evaluated by the proportion of individuals recommended PrEP by the policy- namely, the proportion of iPrEx participants with demographic and risk behaviour characteristics that would yield a PrEP recommendation under the policy, estimated by pooling across the two treatment arms. This metric is a proxy for the resource-utilization of the policy. Second, we evaluated the expected cumulative HIV infection rate under the policy empirically: the Kaplan-Meier method [66] was used to estimate the HIV infection rate among iPrEx participants in the PrEP arm who would be recommended PrEP by the policy, and to estimate the HIV rate among iPrEx participants in the placebo arm who would not be recommended PrEP by the policy. The two estimates were combined using a weighted average, where the weights were determined by the proportion of iPrEx participants who would be recommended PrEP by the policy. Empirical estimation of policy impact is appealing in that it does not require modeling assumptions, and it is made possible by the randomized and placebo-controlled nature of the iPrEx design. Specifically, the randomized trial design ensures that the difference in HIV incidence between the PrEP and placebo groups can be attributed to PrEP itself- rather than to differences in risk taking behaviors or exposure to HIV between the groups. Point estimates and bootstrap-based confidence intervals were bias-corrected to account for having used the same data to predict risk/PrEP benefit and to evaluate population impact. See S1 Methods for details.

## Results

### Univariate associations with HIV infection and PrEP efficacy

Most iPrEx participants [1, 67] were cisgender male (87%) and aged 18–24 (50%) (Table 2). The most frequently reported risk behaviours were insertive or receptive condomless anal intercourse (86%) and more than 5 male sex partners (56%) over the last 3 months. The strongest univariate predictors of increased HIV risk without PrEP were cocaine use over the last month (hazard ratio (HR) 2.58 [95% CI: 1.19–5.62]) and condomless intercourse over the last 3 months (HR 1.23 [95% CI: 0.32–4.62] for insertive only; HR 4.14 [95% CI: 1.28–13.4] for receptive only; 5.11 [95% CI: 1.56–16.74] for receptive and insertive). These variables were also the strongest univariate predictors of increased PrEP efficacy, although, notably, none were statistically significant modifiers of efficacy (Table 2).

**Table 2:** Distributions of demographic and risk behavior variables by treatment arm and their univariate associations with HIV infection risk and PrEP efficacy. Estimated HIV incidence per 100 person-years is reported. The hazard ratio (“risk factor HR”) for each variable quantifies the association between the variable and HIV infection risk, within each treatment arm. The PrEP HR (FTC-TDF vs. Placebo) quantifies the efficacy of PrEP for each level of each variable. The ratio of PrEP HRs quantifies the association between the variable and PrEP efficacy. A Wald test of interaction is reported for each variable.

		Placebo (N = 1218)				FTC-TDF (N = 1224)						
		N	Infections (N)	HIV Incidence	Risk Factor HR (95% CI)	N	Infections (N)	HIV Incidence	Risk Factor HR (95% CI)	PrEP HR	Ratio of PrEP HRs (95% CI)	P-value for Interaction
Gender	Cisgender male	1060	73	0.04	-	1063	38	0.02	-	0.52	-	0.183
	Transgender female	158	10	0.04	0.9 (0.46, 1.74)	161	10	0.04	1.69 (0.84, 3.4)	0.96	1.88 (0.72, 4.91)	
Age	18–24	644	47	0.04	-	578	30	0.03	-	0.72	-	0.202
	25–29	234	15	0.04	0.88 (0.49, 1.57)	266	11	0.02	0.75 (0.37, 1.49)	0.62	0.85 (0.35, 2.1)	
	> = 30	340	21	0.04	0.82 (0.49, 1.38)	380	7	0.01	0.34 (0.15, 0.78)	0.3	0.42 (0.16, 1.1)	
Education	Secondary or less	678	42	0.04	-	693	30	0.02	-	0.7	-	0.169
	Post-secondary	529	41	0.05	1.3 (0.85, 2.01)	515	17	0.02	0.79 (0.44, 1.44)	0.42	0.61 (0.29, 1.27)	
	Missing	11				16				-		
Race	White	201	9	0.03	-	221	3	0.01	-	0.3	-	0.790
	Black/ African American	93	6	0.05	1.54 (0.55, 4.32)	112	4	0.03	2.98 (0.67, 13.31)	0.58	1.94 (0.31, 11.94)	
	Mixed/ Other	857	65	0.04	1.33 (0.66, 2.68)	828	39	0.02	2.55 (0.78, 8.27)	0.61	1.91 (0.49, 7.53)	
	Asian	67	3	0.04	1.4 (0.38, 5.2)	63	2	0.03	3.31 (0.55, 19.92)	0.71	2.36 (0.26, 21.79)	
Cocaine use in past month	None	1165	76	0.04	-	1147	47	0.02	-	0.63	-	0.067
	Cocaine	53	7	0.1	2.58 (1.19, 5.62)	77	1	0.01	0.38 (0.05, 2.74)	0.09	0.15 (0.02, 1.23)	
HIV-positive partner in last 3 months	None	1109	76	0.04	-	1122	45	0.02	-	0.59	-	0.708
	> 1	109	7	0.04	1.14 (0.52, 2.47)	102	3	0.02	0.92 (0.29, 2.98)	0.47	0.81 (0.2, 3.31)	
Unprotected sex in last 3 months	None	174	3	0.01	-	184	6	0.02	-	1.92	-	0.143
	Insertive only	310	8	0.01	1.23 (0.32, 4.62)	317	8	0.01	0.63 (0.22, 1.83)	1.04	0.52 (0.09, 2.82)	
	Receptive only	430	41	0.05	4.14 (1.28, 13.4)	437	17	0.02	0.86 (0.34, 2.19)	0.54	0.21 (0.05, 0.93)	
	Receptive and insertive	304	31	0.06	5.11 (1.56, 16.74)	286	17	0.03	1.43 (0.56, 3.64)	0.41	0.28 (0.06, 1.27)	
Number of male sexual partners in last 3 months	1	98	5	0.03	-	108	4	0.02	-	0.73	-	0.892
	2 to 5	450	26	0.03	1 (0.38, 2.6)	411	11	0.02	0.71 (0.23, 2.23)	0.5	0.71 (0.16, 3.17)	
	> 5	670	52	0.04	1.28 (0.51, 3.2)	705	33	0.03	1.05 (0.37, 2.98)	0.58	0.83 (0.21, 3.31)	
Any transactional sex in last 6 months	No	721	49	0.04	-	717	24	0.02	-	0.49	-	0.339
	Yes	497	34	0.04	0.88 (0.57, 1.37)	507	24	0.03	1.22 (0.69, 2.16)	0.7	1.39 (0.68, 2.84)	
Any self-reported STI in last 6 months	No	910	54	0.04	-	891	33	0.02	-	0.63	-	0.466
	Yes	308	29	0.05	1.37 (0.87, 2.16)	333	15	0.02	1.02 (0.55, 1.87)	0.47	0.74 (0.35, 1.58)	
Seropositive for syphilis at baseline	No	1056	65	0.04	-	1057	40	0.02	-	0.62	-	0.427
	Yes	162	18	0.06	1.64 (0.97, 2.78)	167	8	0.03	1.12 (0.52, 2.4)	0.42	0.68 (0.27, 1.71)	

CI, confidence interval; HR, hazard ratio; PrEP, pre-exposure prophylaxis; STI, sexually transmitted infection; FTC-TDF, emtricitabine/tenofovir disoproxil fumarate

### Model for risk of HIV without PrEP

**Fig 1:**
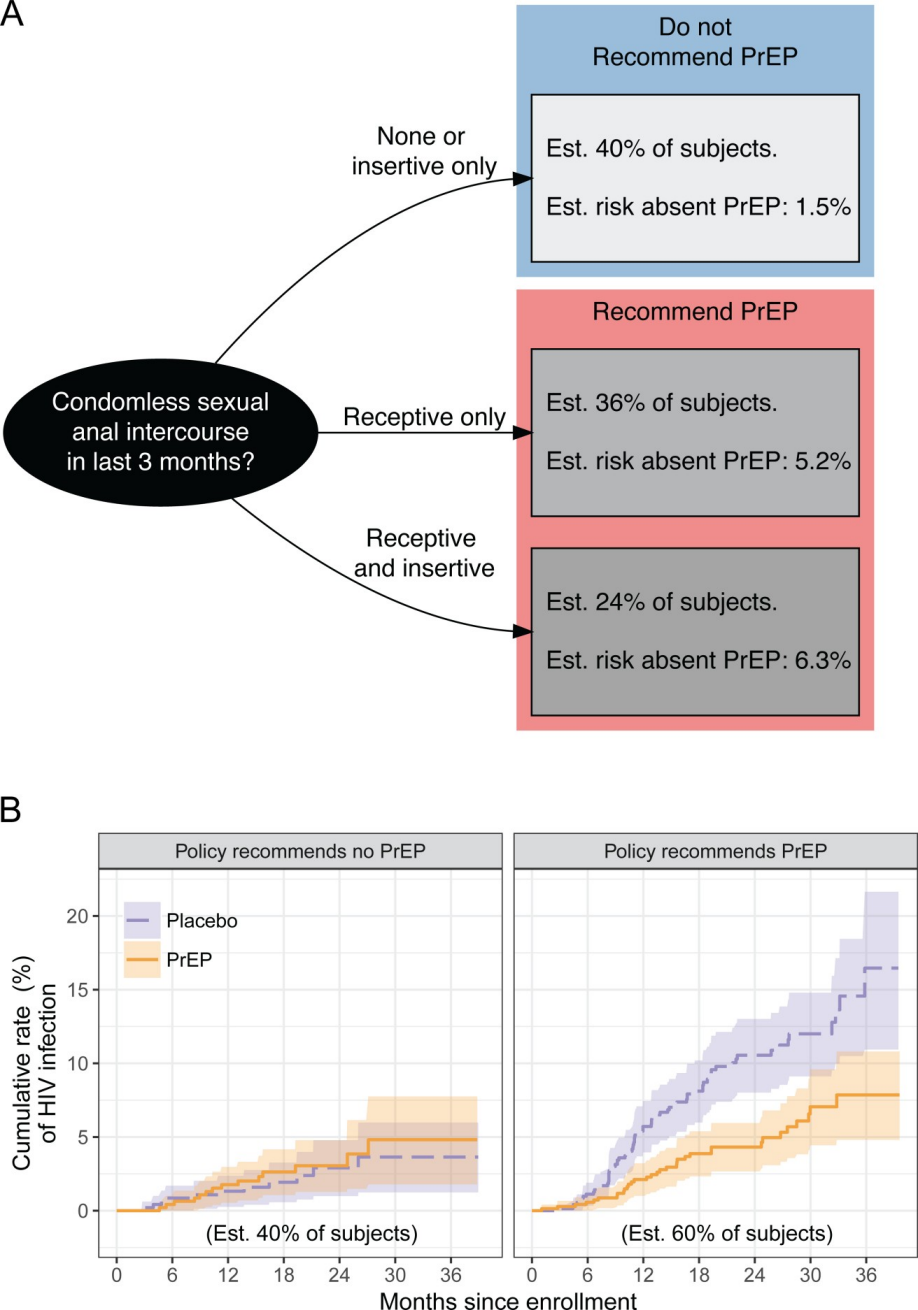
Risk-based PrEP policy and HIV infection risk by PrEP recommendation. Flowchart for determining PrEP recommendation for an individual MSM/TGW under the risk-based policy, which is based on a model for HIV infection risk without PrEP fit to the iPrEx data (A). Empirical estimates of the size of each subpopulation and of the 1-year HIV infection risk without PrEP in each subpopulation are also shown. The PrEP-benefit policy developed using the iPrEx data and using a PrEP benefit threshold of 1.2% is identical. Cumulative rate of HIV infection over time, by treatment arm and risk-based PrEP recommendation, with pointwise 95% confidence intervals (B).

The best Cox proportional hazards logic regression model for predicting HIV infection risk without PrEP is shown in Table 3. Individuals who report engaging in condomless receptive anal intercourse over the last 3 months, without insertive (HR = 3.59 [95% CI: 1.84–6.98]) or with insertive anal intercourse (HR = 4.43 [95% CI: 2.23–8.81]) were estimated to be at considerably increased risk. As shown in Fig 1A, individuals reporting either behaviour were estimated to have a 1-year HIV infection risk above 3%, and were thus recommended PrEP under the risk-based policy (59.7% of individuals); the remaining 40.3% of individuals were not recommended PrEP under this policy. The risk model itself was reasonably stable across bootstrap samples; the condomless receptive intercourse and condomless receptive and insertive intercourse variables were selected in 342 and 364 of the 500 models fit to bootstrapped datasets, respectively (S1 Fig). More importantly, the risk-based PrEP recommendations were highly stable, with 40% of individuals recommended PrEP in 70% or more bootstrap samples and the other 60% not recommended PrEP in 89% or more samples (S4 Fig).

**Table 3:** The best Cox proportional hazards logic regression models for predicting HIV infection risk without PrEP (fit using placebo arm data) and with PrEP (fit using FTC-TDF arm data). For each baseline demographic or risk behaviour variable entering in the model, the associated hazard ratio (HR) for HIV infection is shown.

Without PrEP (Placebo Arm)			With PrEP (FTC-TDF Arm)		
Baseline demographic/risk behaviour variable	HR (95% CI)	p-value	Baseline demographic/risk behaviour variable	HR (95% CI)	p-value
Condomless receptive only anal intercourse in last 3 mo.	3.59 (1.84–6.98)	0.0002	Younger than 30 yrs.	2.78 (1.25–6.21)	0.013
Condomless receptive and insertive anal intercourse in last 3 mo.	4.43 (2.23–8.81)	< 0.0001	Condomless receptive and insertive anal intercourse in last 3 mo.	1.87 (1.04–3.40)	0.037

CI, confidence interval; HR, hazard ratio; PrEP, pre-exposure prophylaxis; FTC-TDF, emtricitabine—tenofovir disoproxil fumarate.

For comparison, we developed risk models using alternative model-building and machine-learning approaches. The best stepwise Cox proportional hazards regression model was identical to that built using Cox logic regression (S1 Table) and had similar population impact; small differences appeared between models in bootstrapped datasets (S2 Table and S2 Fig). Using Cox regression model with lasso, the best-predicting model involved many more variables (S1 Table) but had a similar estimated impact (S2 Table) and was much less stable across bootstrap samples (S3 and S4 Figs).

### Model for PrEP benefit

The best Cox proportional hazards logic regression model for predicting HIV infection risk with PrEP is shown in Table 3. Individuals under age 30 (HR 2.78; 95% CI: 1.25–6.21) and who reported condomless receptive and insertive anal intercourse over the last 3 months (HR 1.87; 95% CI: 1.04–3.40) were estimated to be at increased risk with PrEP. By combining this with the model for HIV risk without PrEP, a model for PrEP benefit as a function of age and condomless intercourse was obtained. However, although age predicts some variation in the level of PrEP benefit (S4 Fig), only condomless intercourse determines the PrEP recommendation under the PrEP-benefit-based policy with a benefit threshold of 1.2%: individuals reporting condomless receptive or insertive and receptive anal intercourse are predicted to have at least a 1.2% reduction in 1-year HIV infection risk due to PrEP, and are recommended PrEP under the policy (59.7% of individuals). Importantly, therefore, this PrEP-benefit-based policy recommends PrEP to the same subpopulation as does the risk-based policy.

We explored the use of an alternative PrEP benefit threshold; few thresholds could be examined given that the PrEP benefit model only predicts six levels of PrEP benefit (for three levels for type of condomless intercourse and two levels of age). Using a lower PrEP benefit threshold of 0.7%—corresponding to a 0.7% lower 1-year risk of HIV with PrEP and an NNT of 143—would result in recommending PrEP to individuals who report condomless receptive or insertive and receptive anal intercourse or who are 30 years or older (S4 Fig), an estimated 71% of the iPrEx population.

We found that the PrEP benefit model and associated PrEP-benefit-based PrEP recommendations were less stable across bootstrap samples than their risk-based counterparts (S5 Fig). Alternative modelling approaches did not yield policies with improved performance (S2 Table).

### Population impact of PrEP policies

Fig 1B shows the estimated cumulative rates of HIV infection over time by treatment arm, for subpopulations of MSM/TGW who would or would not be recommended PrEP under the risk-based policy. The policy is estimated to achieve a 1.95% 1-year HIV infection rate (95% CI: 1.21%-2.73%), below the 1.97% achieved if PrEP is recommended to all MSM/TGW in the iPrEx population (95% CI: 1.16%-2.86%) (Table 4). Strikingly, the policy would require treating just 59.7% of MSM/TGW (95% CI: 24.9%-100%). The benefit of PrEP in the high-risk subgroup is an absolute 3.31% reduction in 1-year HIV incidence (95% CI: 1.20%-6.12%), corresponding to an NNT of 30 –as opposed to a policy of PrEP for all MSM/TGW which has an NNT of 49.

**Table 4:** Estimated impact of risk-based, PrEP benefit-based, and CDC PrEP policies for the MSM/TGW population. Policies are ordered by the associated proportion of the population that is recommended PrEP. Impact is shown over 1 and 2 years post-enrolment.

	Proportion recommended PrEP (95% CI)	Reduction in HIV incidence in subpopulation recommended PrEP (95% CI)	HIV incidence under policy (95% CI)	Reduction in HIV incidence in subpopulation recommended PrEP (95% CI)	HIV incidence under policy (95% CI)
		1 year post-enrolment		2 years post-enrolment	
**PrEP for none**	0%	--	4.01	--	7.75
			(2.89–5.08)		(5.95–9.50)
**Risk-based**	59.7	3.31	1.95	5.53	3.83
**policy**	(24.9–100)	(1.20–6.12)	(1.21–2.73)	(2.55–12.8)	(2.67–5.43)
**PrEP-benefit-**	59.7	3.11	2.07	5.30	4.05
**based policy (1.2%**	(26.1–95.5)	(1.45–5.50)	(1.28–3.10)	(2.58–8.83)	(2.84–6.62)
**threshold)**					
**PrEP-benefit-**	71.0	2.84	1.94	4.30	4.36
**based policy (0.7%**	(36.7–100)	(1.37–5.12)	(1.18–2.76)	(2.40–8.77)	(2.75–5.82)
**threshold)**					
**CDC guideline**	86.4	2.32	1.97	4.04	4.47
	(85.0–87.6)	(0.75–3.92)	(1.11–2.91)	(1.83–6.29)	(3.00–6.15)
**PrEP for all**	100%	2.04	1.97	3.91	3.84
		(0.66–3.55)	(1.16–2.86)	(1.62–6.03)	(2.57–5.10)

CDC, Centers for Disease Control and Prevention; CI, confidence interval; PrEP, pre-exposure prophylaxis.

Table 4 also shows the estimated population impact of the PrEP-benefit-based policy that uses a PrEP benefit threshold of 1.2%. Since this PrEP-benefit-based policy is identical to the risk-based policy, the estimated impact of the two policies is similar; minor differences between the models occur in some bootstrap samples, with the latter model being more variable. Using a lower PrEP benefit threshold of 0.7% would result in more individuals being recommended PrEP (71.0% vs. 59.7%) and similar HIV infection rates at 1 and 2 years.

The estimated impact of the CDC guideline is shown in Table 4 as well. This guideline is estimated to recommend PrEP to a larger subpopulation of MSM/TGW than the risk-based policy (86.4% vs. 59.7%), and yet it is estimated to achieve a very similar estimated 1-year HIV infection rate (1.97% vs. 1.95%). However, it should be noted that the confidence interval for the proportion of the population to be recommended PrEP under the risk-based policy is wide, and does not rule out the possibility that the policy recommends PrEP to the same-sized subpopulation as the CDC guideline. The confidence intervals for risk- and PrEP-benefit based policies, which are derived using the iPrEx data, are wide because they account for the uncertainty in the risk- and PrEP-benefit models. In contrast, the CDC policy is fixed, having been derived using historical data, and therefore the size of the subpopulation to be recommended PrEP is estimated much more precisely. Separate data will be needed to validate the apparent difference in resource-utilization of the risk- and CDC policies.

### Contrasting PrEP policies

**Fig 2:**
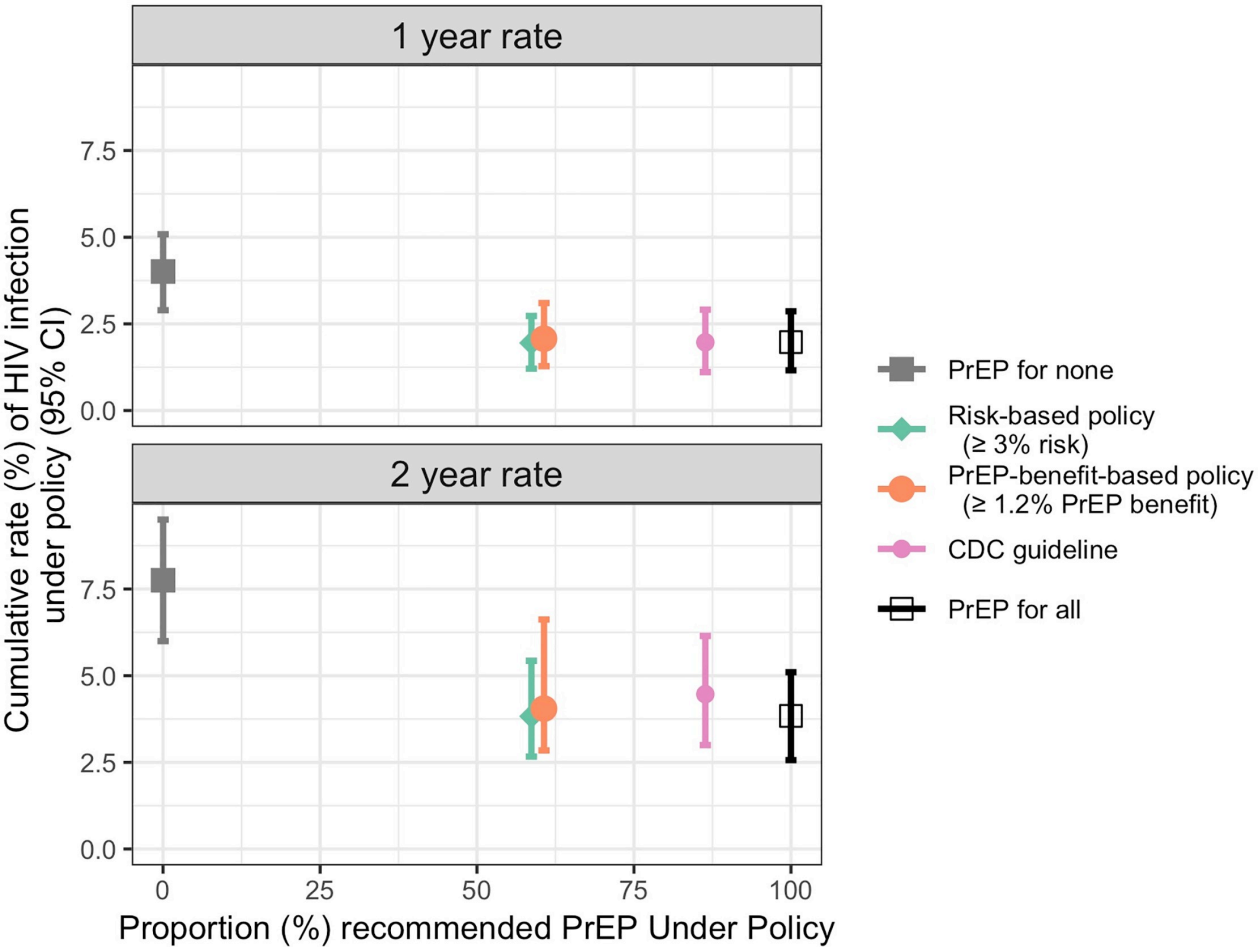
Contrasting PrEP policies with a policy that recommends PrEP to all individuals. Policies are contrasted in terms of the proportion of individuals recommended PrEP by the policy (x-axis) and the estimated 1- and 2-year HIV infection rates under the policy (y-axis). Symbols show the estimated 1- and 2-year infection rates and lines show 95% confidence intervals.

Although a PrEP-benefit-based policy has theoretical appeal, the risk-based policy and PrEP-benefit-based policy (1.2% threshold) that we developed using the iPrEx data were found to be identical, and therefore have similar estimated population impact (Table 4). Fig 2 highlights this visually, showing the estimated 1- and 2-year HIV infection rates achieved using the policies and the estimated rate under a policy that recommends PrEP to all. The very similar performance of the risk- and PrEP-benefit-based policies reflects the fact that there are not strong interactions between PrEP and baseline demographic and risk behaviour variables, and suggests that an individual’s risk of HIV without PrEP is all that is needed to identify individuals with high absolute reduction in HIV risk due to PrEP. Coupled with the fact that the risk-based policy is more stable across bootstrap samples, we view the risk-based policy as having greater potential.

These results do identify an important difference between the risk-based policy optimized using iPrEx data and the CDC guideline. While both policies achieve nearly the same HIV incidence as PrEP for all MSM/TGW, the CDC guideline is estimated to recommend PrEP to a larger subpopulation of MSM/TGW (Fig 2). An estimated 28.9% of individuals would be recommended PrEP by the CDC guideline but not by the risk-based policy, and another 2.2% would not be recommended PrEP by the CDC guideline but would by the risk-based policy (S3 Table). Importantly, the CDC guidelines are based on previous studies of HIV risk factors among US MSM in HIV prevention trials [59–61]. These results suggest that the CDC guidelines may be broader than they need to be to achieve a substantial reduction in HIV incidence.

It may be of interest to compare the HIV incidence achieved under policies that are constrained to use the same resources, i.e. to treat a subpopulation of the same size. However, such policies are difficult to examine with the risk and PrEP benefit models fit to the iPrEx data; the fitted risk model takes only three levels and the PrEP benefit model takes six levels and therefore the size of the subpopulations treated cannot be controlled with precision. In particular, employing anything lower than the 3% high risk threshold we used would mean PrEP is recommended to the entire MSM/TGW population. Using a lower PrEP benefit threshold of 0.7% would result in a PrEP-benefit-based policy that recommends PrEP to a similar fraction of the population as does the CDC guideline (71% vs. 86%), and the estimated HIV incidences under these two policies are highly similar (Table 3).

**Fig 3:**
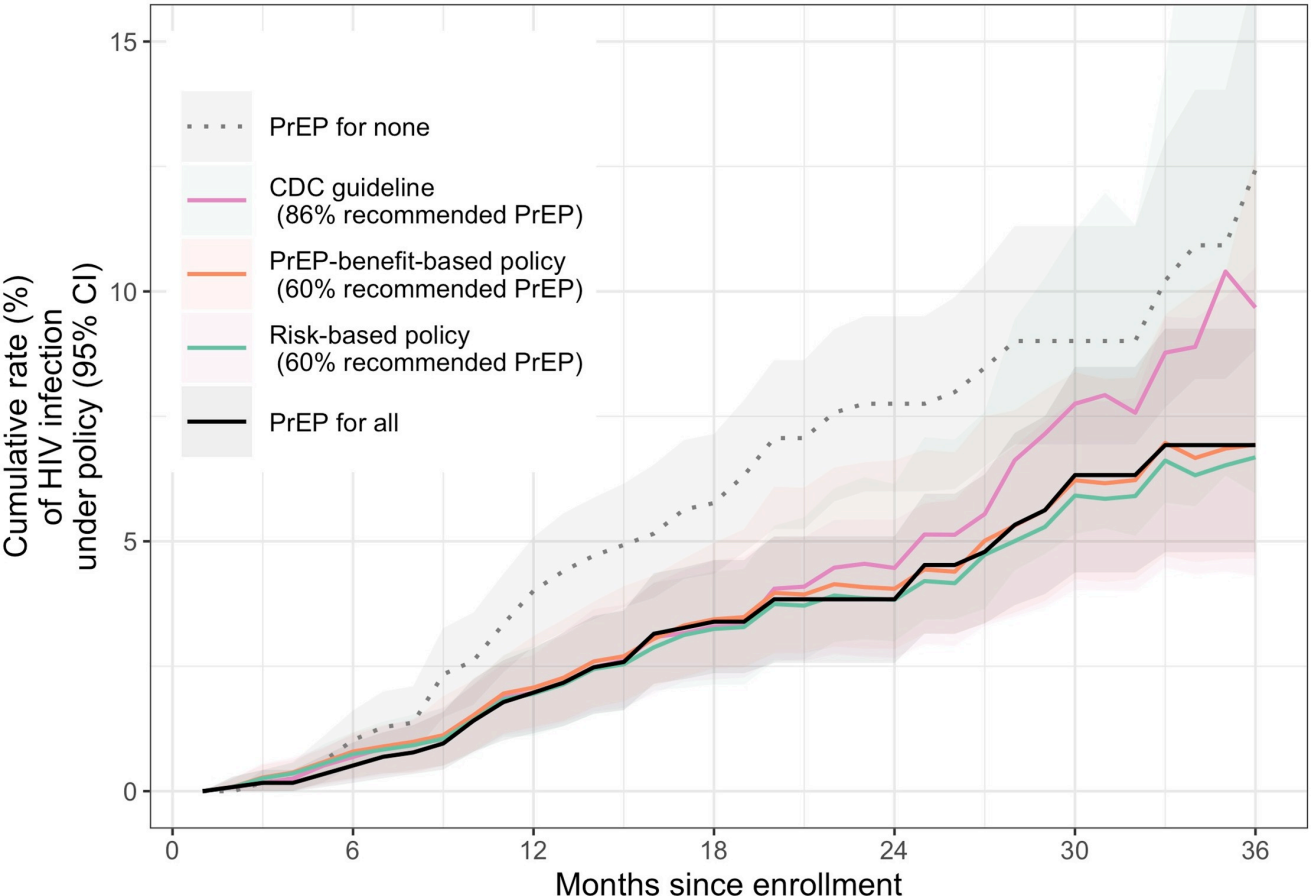
HIV infection risk over time under risk-based, PrEP-benefit based, and CDC PrEP policies. Cumulative rate of HIV infection over time with pointwise 95% confidence intervals. The PrEP-benefit-based policy uses a PrEP benefit threshold of 1.2%.

Point estimates suggest that all of these PrEP policies may have declining impact over time (Fig 3), especially the CDC guideline. This result is somewhat expected, given that the predictive capacity of the baseline risk behaviour variables may diminish with time.

### Population impact under higher adherence

Data suggest that adherence to PrEP may be higher in "real world" contexts, where individuals know that PrEP is effective and that they are in fact receiving it, as opposed to being blinded to PrEP vs. placebo receipt, as in the iPrEx study [4, 5, 43–50]. In addition, many analyses have shown that PrEP efficacy is strongly associated with adherence [51, 68–70]. Therefore, it is of interest to determine whether the impact of the PrEP policies we examine would differ in settings with higher adherence. We conducted a simple sensitivity analysis to address this question. Specifically, we examined scenarios where we assumed that the relative risk associated with PrEP was reduced by a factor of 1.0 to 0.1, due to improved adherence relative to that seen in the iPrEx study. This corresponds to varying the overall PrEP relative-risk from 0.56 (the observed relative risk) to 0.06. A key limitation of this sensitivity analysis is that there are no data we know of to inform on whether the same decrease in PrEP relative risk would apply equally to all subgroups of the MSM/TGW population, or whether some subgroups would have greater decreases in PrEP relative risk than others due to better adherence. Because neither study-level meta-analyses associating efficacy with adherence [69, 70] nor analyses of efficacy among adherers in individual trials [51, 68] inform on this, for simplicity we assume that the same multiplicative decrease in PrEP relative risk applies to the iPrEx population at large, as well as to the high risk subgroup identified by our risk-based PrEP policy and the high risk subgroup identified by the CDC policy.

**Fig 4:**
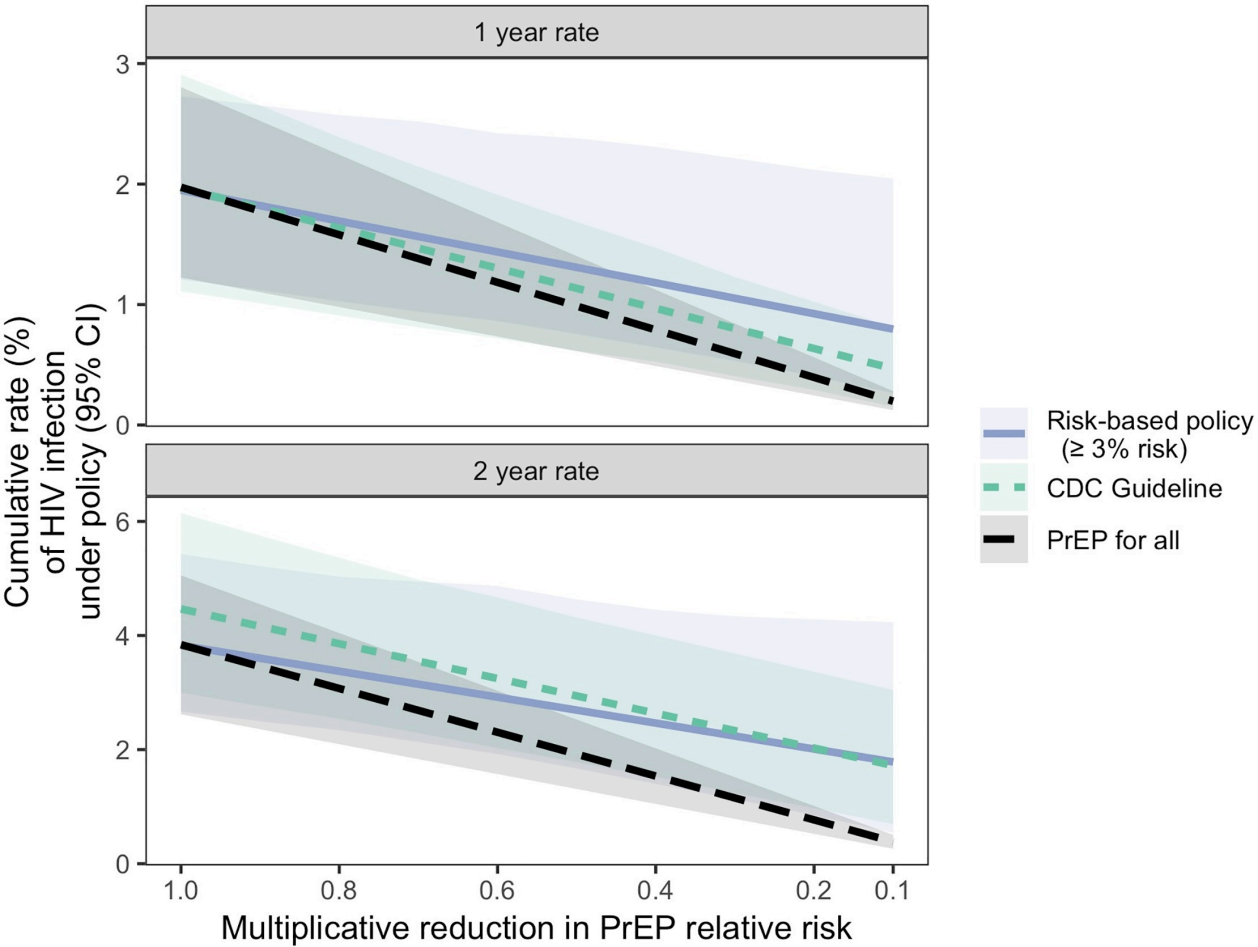
Sensitivity analysis: HIV infection rate under PrEP for all, risk-based, and CDC PrEP policies, with varying PrEP relative risk. Cumulative 1- and 2-year HIV infection rates under each policy, as a function of the multiplicative reduction in PrEP relative risk due to increasing adherence. An 0.9 multiplicative reduction in PrEP relative risk reduces the relative risk from 0.56 to 0.50. Pointwise 95% confidence intervals are shown with shading.

Fig 4 shows the results of this sensitivity analysis. We make two observations. First, as expected, as the PrEP relative risk decreases, the HIV infection rate achieved under all policies decreases. Second, as the PrEP relative risk decreases, the PrEP for all policy has a more rapid decline in HIV infection rate than do the risk-based and CDC policies. This is because the latter two policies recommend PrEP to just 59.7% and 86.4% of the MSM/TGW population, respectively, and so the reduction in HIV due to PrEP only affects these subpopulations. Note that the size of the subpopulations recommended PrEP does not change across the scenarios examined here. These results suggest that risk-based policies may have less appeal in settings with higher adherence. We caution, however, that these results are a direct consequence of our assumption that the multiplicative reduction in PrEP relative risk is the same across all subpopulations. A more comprehensive modeling approach- if informed by data on how efficacy changes as a function of adherence in different subgroups of the population- could more effectively compare the impact of different PrEP policies while allowing for different patterns of adherence.

## Discussion

We analysed data from a landmark multi-national PrEP efficacy study in MSM/TGW to identify subpopulations predicted to be at high HIV risk without PrEP and subpopulations with high expected PrEP benefit. Based on these models, we defined risk-based and PrEP-benefit based policies for prioritizing PrEP, and evaluated and compared the policies empirically, in terms of the size of the subpopulation recommended PrEP under each policy and the expected HIV incidence under each policy. We found the risk- and PrEP-benefit-based policies to have similar estimated impact in the MSM/TGW population in iPrEx, consistent with our analyses and prior work suggesting that the PrEP effect was relatively constant on the relative risk scale, i.e. there was no strong effect modification [1, 67]. We compared the risk-based policy derived using the iPrEx data to the CDC PrEP guideline for MSM, and estimated that it would prioritize PrEP to a smaller subpopulation while achieving a similar reduction in HIV incidence. Risk-based prioritization of PrEP appears to be a resource-efficient strategy for resource-limited settings, achieving nearly the same reduction in HIV incidence as does rolling out PrEP for all MSM/TGW.

Critically, our results pertain directly only to the MSM/TGW population from which iPrEx participants were recruited. The demographic and risk behaviours that best predict HIV risk may differ in other populations. The impact of the iPrEx-derived risk-based policy may not generalize to other populations, either because the distribution of demographic and risk characteristics differs, or because the level of PrEP efficacy differs. For example, recent studies of Black American MSM and young MSM have found HIV incidence exceeding the iPrEX rates [71, 72]. In the PROUD open-label PrEP study [5], PrEP efficacy was estimated at 86%, much higher than in iPrEx. As illustrated by our sensitivity analysis, in settings where larger subpopulations are high risk and prioritized for PrEP, or where PrEP efficacy is higher, we may expect to see smaller resource savings of a risk-based PrEP policy as compared to a policy of PrEP for all.

Given that the iPrEx study was an individually-randomized trial, these results only characterise the impact of PrEP policies attributable to the direct effect of PrEP (as opposed to the total effect [73]). The duration of iPrEx follow-up also only permits estimation of impact over 1–3 years of follow-up. The reliability of the risk behaviour variables may also differ in iPrEx as opposed to more routine clinical settings [74–76].

The risk-based PrEP policy optimized using iPrEx data would recommend PrEP to MSM/TGW who report engaging in condomless receptive anal intercourse, or condomless receptive and insertive anal intercourse. However, a meaningful benefit of PrEP cannot be ruled out for MSM/TGW who engage in exclusively insertive anal intercourse. For example, there may be individual factors, such as an HIV-infected partner who is not virally suppressed, that would clearly lend themselves to a recommendation for PrEP. Providers must base their prescribing practices on individual- rather than population-level impact.

Mathematical modelling is and will continue to be essential for PrEP policy research. Using mathematical models allows researchers to study and isolate the influence of factors such PrEP uptake and adherence on population impact. Modeling can also integrate multiple sources of data, e.g. population distributions of demographic and risk behaviours, as opposed to distributions among individuals eligible for and willing to enrol in clinical trials. Modeling can capture both direct and indirect effects of PrEP, examine impact over longer time periods, and formally incorporate assumptions about the cost of providing PrEP. However, model-based estimates of PrEP impact are only as reliable as their data inputs and underlying assumptions. Mathematical models of PrEP impact typically assume the existence of subpopulations with different behaviors and levels of HIV risk, that PrEP reduces risk by a factor that is constant across risk groups, and that adherence increases PrEP efficacy by a constant amount across risk groups. We posit that PrEP efficacy trials, which have limited generalizability but which enable population impact to be estimated directly using observed data, can highlight policies for further investigation and provide preliminary estimates of population impact, thus complementing the modelling and contributing to policy discussions.

Buchbinder and colleagues [67] previously analysed the iPrEx trial data to assess the baseline demographic and risk behaviour variables individually for their ability to predict HIV infection risk without PrEP (in the placebo arm), and the population attributable fraction and NNT were calculated for risk behaviour subgroups. Two variables, condomless insertive or receptive anal intercourse, and condomless receptive anal intercourse with a partner of unknown HIV serostatus, were identified as being most important for prioritizing PrEP rollout. Our analyses went further to build multivariate models to predict not only HIV infection risk without PrEP, but also to model PrEP benefit as a function of demographic and risk behavior variables. The first variable identified by [67], but not the second, was selected into our multivariate risk- and PrEP-benefit models, and forms the basis for our associated risk-based PrEP policy. We also evaluated PrEP policies based on our multivariate risk- and PrEP-benefit models, using measures that directly characterize the population impact of the policies: the proportion of the population recommended PrEP, and the reduction in HIV incidence under the policy.

Zheng et al. [77] recently put forth statistical methods for developing PrEP policies for resource-limited settings, based on criteria for maximizing the proportion of would-be HIV-infected subjects absent PrEP who are identified and recommended PrEP (i.e. sensitivity) subject to a cost constraint (fraction of population treated), or based on minimizing cost subject to a fixed sensitivity. Policies were evaluated in terms of sensitivity and the number needed to test to detect one HIV infection, using survey data from Eastern Uganda. Instead, given randomized trial data, we evaluate policies in terms of their impact on HIV incidence. Furthermore, the risk- and benefit-based policies we consider are grounded in decision theory and are designed to maximize the net benefit of a policy.

## Conclusions

We conclude that risk-based policies that prioritize PrEP for MSM/TGW subpopulations at highest risk of HIV without PrEP are worth further investigation for resource-limited settings. Risk-based policies are easy to understand and interpret and we did not find greater impact of policies that prioritize based on expected PrEP benefit. The existing CDC guideline, which requires measurement of 7 demographic and risk factors including number of male partners and condomless intercourse, or our more parsimonious risk model that is based only on condomless intercourse, could be the focus of future policy research for the MSM and TGW population. Our statistical approach could be used to explore and evaluate PrEP policies for other populations.

## Supporting information

S1 Methods Supplementary methods. (DOCX)

S1 Table Cox proportional hazards regression models, selected using stepwise model selection using the Lasso penalty, for predicting HIV infection risk without PrEP (fit using placebo arm data) and with PrEP (fit using FTC-TDF arm data). For each baseline demographic or risk behaviour variable entering in the model, the associated hazard ratio (HR) for HIV infection is shown.
(DOCX)

S2 Table Estimated population impact of risk-based and PrEP-efficacy-based PrEP policies, over 1 year. Policies are based on risk and PrEP-benefit Cox proportional hazards regression models built using stepwise and lasso model selection methods. PrEP-benefit-based policies use a PrEP benefit threshold of 1.2%. Shown for comparison is the impact of a policy based on the HIRI-MSM risk score which is discussed in the US CDC PrEP guidelines and which recommends PrEP to individuals with HIRI-MSM risk scores of 10 or more.* Policies are ordered by the associated proportion of the population that is recommended PrEP.
(DOCX)

S3 Table Comparison of PrEP recommendations for risk-based PrEP policy and US CDC PrEP guideline. (DOCX)

S1 Fig Variable importance summary for Cox proportional hazards logic regression models. Bar charts show how many times a variable was selected across 500 bootstrap datasets.
(DOCX)

S2 Fig Variable importance summary for Cox proportional hazards regression stepwise models. Bar charts show how many times a variable was selected across 500 bootstrap datasets.
(DOCX)

S3 Fig Variable importance summary for Cox proportional hazards regression with lasso models. Bar charts show how many times a variable was selected across 500 bootstrap datasets.
(DOCX)

S4 Fig Flowchart for determining PrEP recommendation for an individual MSM/TGW under the PrEP-benefit-based policy, which is based on a model for PrEP benefit fit to the iPrEx data. A PrEP benefit threshold of 1.2% is used. Empirical estimates of the size of each subpopulation and of the reduction in 1-year HIV infection risk due to PrEP are shown. The PrEP benefit model describes gradients in PrEP benefit as a function of age, within condomless intercourse subgroups.
(DOCX)

S5 Fig Distribution of the proportion of times each individual is recommended PrEP across 500 bootstrap datasets, for policies of different types (risk- and PrEP-benefit based policies built using different modelling approaches). A policy that has more mass near 0 and 1 produces more stable treatment recommendations. PrEP-benefit-based policies use a PrEP benefit threshold of 1.2%.
(DOCX)
