# Supplementary material for: Evaluating the impact of policies recommending PrEP to subpopulations of men and transgender women who have sex with men based on demographic and behavioral risk factors
Source: PLoS One. 2019 Sep 19;14(9):e0222183. doi: 10.1371/journal.pone.0222183 (PMC6752862; doi:10.1371/journal.pone.0222183)
Supplement: S1 Methods — (DOCX) [file pone.0222183.s001.docx]

**S1 Methods**

**Details of logic regression modelling**

Cox proportional hazards logic regression uses a simulated annealing algorithm to sift through all Boolean combinations of binary predictors to maximize the Cox partial likelihood. There are two tuning parameters involved: 1) the total number of Boolean combinations of variables (‘trees’) in each model; and 2) the total number of variables included in the model (‘leaves’). We used 10-fold cross-validation to determine the choice of number of trees (1-5 considered) and number of leaves (1-5 considered) for models in each treatment arm. For both arms, this suggested fitting models with a maximum of 2 trees and 2 leaves.

**Other approaches for modelling risk and PrEP benefit**

We employed two other modelling-building strategies: Cox proportional hazards regression with stepwise variable selection, and Cox proportional hazards regression with Lasso penalty. The Bayesian information criterion (BIC) was used for the forward/backward stepwise approach because it yielded models of similar size as those selected using logic regression—it produced a median of two covariates selected across bootstrap replicates. For the Cox Lasso method, 10-fold cross-validation was used to select the penalty. Cox proportional hazards models were re-fit using the Lasso-selected variables.

**HIRI-MSM risk score calculation**

In addition to the recommended indications for PrEP use among MSM laid out in the US CDC PrEP guidelines (S3 Table), there is also suggestion to use a previously-published HIV risk score (HIRI-MSM), which is a function of 7 demographic and behavioural risk factors including age and unprotected intercourse [1], as the basis for determining indications for PrEP in the MSM population. Individuals with scores of 10 or more are deemed high risk and recommended PrEP [2]. We calculated HIRI-MSM risk scores for each iPrEx participant as best as possible, given the available data. Below we contrast calculation of the original HIRI-MSM score vs. our approximate score.

1. How old are you today?
   1. HIRI-MSM score
      1. If < 18 years, score 0.
      2. If 18-28 years, score 8.
      3. If 29-40 years, score 5,
      4. If 41-48 years, score 2,
      5. If 49 years or more, score 0
   2. Adapted score:
      1. If 18-29, score 8
      2. If 30-39, score 5
      3. If 40 years or more, score 2
2. In the last 6 months, how many men have you had sex with?
   1. HIRI-MSM score
      1. If >10 male partners, score 7.
      2. If 6–10 male partners, score 4.
      3. If 0-5 male partners, score 0.
   2. Adapted score: (note our variable pertains to the last 3 months)
      1. If more than 5 partners, score 7
      2. If 2-5 partners, score 4
3. In the last 6 months, how many times did you have receptive anal sex (you were the bottom) with a man without a condom?
   1. HIRI-MSM score
      1. If 1 or more times, score 10, otherwise, score 0
   2. Adapted score: (note our variable pertains to the last 3 months)
      1. If 1 or more times, score 10, otherwise, score 0
4. In the last 6 months, how many of your male sex partners were HIV-positive?
   1. HIRI-MSM score
      1. If >1 positive partner, score 8
      2. If 1 positive partner, score 4.
   2. Adapted score: (note our variable pertains to the last 3 months)
      1. If >= 1 positive partner, score 4
5. In the last 6 months, how many times did you have insertive anal sex (you were the top) without a condom with a man who was HIV- positive?
   1. HIRI-MSM score
      1. If 5 or more times, score 6.
   2. Adapted score: (note our variable pertains to the last 3 months)
      1. We approximated this using indicators of condomless insertive anal intercourse and intercourse with an HIV-positive partner; participants who reported condomless insertive intercourse and who reported intercourse with an HIV-positive partner and who reported > 2 partners, score 6
6. In the last 6 months, have you used methamphetamines such as crystal or speed?
   1. HIRI-MSM score
      1. If yes, score 6
   2. Adapted score:
      1. We did not have this variable. No score adjustment was done.

**Estimation of population impact**

We estimated empirically the proportion of subjects recommended PrEP under each policy. Kaplan-Meier methods were used to estimate differences in cumulative rates of HIV with vs. without PrEP in the subpopulation recommended PrEP, and to estimate HIV rates in the population under the policy. Specifically, let *D* be an indicator of HIV infection over a specified time frame (1 or 2 years post-enrollment), *T* be an indicator of PrEP vs. Placebo assignment, *X* be a vector of baseline covariates involved in a given policy, and $A\left( X \right)$ be an indicator of the treatment recommended by the policy; $A\left( X \right)$ = 1 indicates a PrEP recommendation and $A\left( X \right)$= 0 does not. We estimate the HIV infection rate under the policy by

$$\hat{P}\left( D=1 \right|T=1, A\left( X \right)=1)\cdot\hat{P}\left( A\left( X \right)=1 \right)+\hat{P}(D=1|T=0,A\left( X \right)=0)\cdot\hat{P}(A\left( X \right)=0)$$

where $\hat{P}\left( A\left( X \right)=a \right)$ is the proportion of participants (pooling the two treatment arms) recommended treatment *a* by the policy, and $\hat{P}\left( D=1 \right|T=a, A\left( X \right)=a)$ is the Kaplan-Meier estimate of the HIV infection rate among participants on arm *a* who are recommended treatment *a*.

We employed the refined bootstrap bias correction method [3] to account for modelling risk and PrEP benefit, and evaluating impact of policies based on these predictions, using the same data. We calculated confidence intervals for the measures using the percentile bootstrap, and bias-corrected the confidence intervals by shifting them down by the estimated bias.

**References**

[1] Smith DK, Pals SL, Herbst JH, Shinde S, Carey JW. Development of a clinical screening index predictive of incident HIV infection among men who have sex with men in the United States. Journal of AIDS. 2012;60(4):421-7.

[2] US Public Health Service. Preexposure prophylaxis for the prevention of HIV infection in the United States: A Clinical Practice Guideline. 2014.

[3] Efron B, Tibshirani R. An Introduction to the Bootstrap: Chapman and Hall/CRC 1994.
