# Supplementary material for: Evaluating the impact of policies recommending PrEP to subpopulations of men and transgender women who have sex with men based on demographic and behavioral risk factors
Source: PLoS One. 2019 Sep 19;14(9):e0222183. doi: 10.1371/journal.pone.0222183 (PMC6752862; doi:10.1371/journal.pone.0222183)
Supplement: S1 Table — For each baseline demographic or risk behaviour variable entering in the model, the associated hazard ratio (HR) for HIV infection is shown. (DOCX) [file pone.0222183.s002.docx]

**Table S1: Cox proportional hazards regression models, selected using stepwise model selection using the Lasso penalty, for predicting HIV infection risk without PrEP (fit using placebo arm data) and with PrEP (fit using FTC-TDF arm data).** For each baseline demographic or risk behaviour variable entering in the model, the associated hazard ratio (HR) for HIV infection is shown.

|  | Without PrEP (Placebo Arm) | | | With PrEP (FTC-TDF Arm) | | |
| --- | --- | --- | --- | --- | --- | --- |
|  | Baseline demographic/risk behaviour variable | HR  (95% CI) | p-value | Baseline demographic/risk behaviour variable | HR  (95% CI) | p-value |
| **Stepwise** |  |  |  |  |  |  |
|  | Condomless receptive anal intercourse in last 3 mo. | 3.59 (1.84 – 6.98) | 0.0002 | 30 yrs. or older | 0.38 (0.17 – 0.84) | 0.017 |
|  | Condomless receptive and insertive anal intercourse in last 3 mo. | 4.43 (2.23 – 8.81) | < 0.0001 |  |  |  |
| **Lasso** |  |  |  |  |  |  |
|  | Transgender | 0.63 (0.31 – 1.26) | 0.19 | Transgender | 1.80 (0.89 – 3.63) | 0.103 |
|  | Cocaine | 2.50 (1.13 – 5.51) | 0.02 | 30 yrs. or older | 0.35 (0.15 – 0.77) | 0.010 |
|  | Condomless insertive anal intercourse in last 3 mo. | 1.24 (0.33 – 4.70) | 0.75 | Condomless receptive and insertive anal intercourse in last 3 mo. | 1.91 (1.05 – 3.47) | 0.035 |
|  | Condomless receptive and insertive anal intercourse in last 3 mo. | 4.88 (1.49 – 15.9) | 0.01 | 2 – 5 sexual partners in last 3 mo. | 0.70 (0.36 – 1.39) | 0.3116 |
|  | Condomless receptive anal intercourse in last 3 mo. | 4.42 (1.36 – 14.4) | 0.01 |  |  |  |
|  | Seropositive for syphilis at baseline | 1.40 (0.82 – 2.37) | 0.21 |  |  |  |
