# Supplementary material for: Evaluating the impact of policies recommending PrEP to subpopulations of men and transgender women who have sex with men based on demographic and behavioral risk factors
Source: PLoS One. 2019 Sep 19;14(9):e0222183. doi: 10.1371/journal.pone.0222183 (PMC6752862; doi:10.1371/journal.pone.0222183)
Supplement: S2 Table — Policies are based on risk and PrEP-benefit Cox proportional hazards regression models built using stepwise and lasso model selection methods. PrEP-benefit-based policies use a PrEP benefit threshold of 1.2%. Shown for comparison is the impact of a policy based on the HIRI-MSM risk score which is discussed in the US CDC PrEP guidelines and which recommends PrEP to individuals with HIRI-MSM risk scores of 10 or more.* Policies are ordered by the associated proportion of the population that is recommended PrEP. (DOCX) [file pone.0222183.s003.docx]

**Table S2: Estimated population impact of additional risk-based and PrEP-efficacy-based PrEP policies, over 1 year.** These policies are based on risk and PrEP-benefit Cox proportional hazards regression models built using alternative approaches-- stepwise or lasso model selection methods. PrEP-benefit-based policies use a PrEP benefit threshold of 1.2%. Shown for comparison is the impact of a policy based on the HIRI-MSM risk score which is discussed in the US CDC PrEP guidelines and which recommends PrEP to individuals with HIRI-MSM risk scores of 10 or more.* Policies are ordered by the associated proportion of the population that is recommended PrEP.

|  | **Proportion recommended PrEP (95% CI)** | **Reduction in 1-yr. HIV incidence in subpopulation recommended PrEP (95% CI)** | **Estimated 1-yr. HIV incidence under policy (95% CI)** |
| --- | --- | --- | --- |
| **PrEP for none** | 0% | -- | 4.01  (2.89 - 5.08) |
| **Risk-based Policy: Stepwise** | 59.7  (28.2 – 75.5) | 3.45  (1.22-5.83) | 1.88  (1.01 - 2.74) |
| **Risk-based Policy: Lasso** | 60.9  (37.5 – 64.9) | 3.53  (1.57 – 5.86) | 1.81  (1.10 – 2.84) |
| **PrEP-benefit: Stepwise** | 59.7  (0.1 – 73.7) | 2.26  (-1.28 – 4.49) | 2.58  (0.78 – 3.43) |
| **PrEP-benefit: Lasso** | 55.3  (34.4 – 71.2) | 1.96  (0.69 – 4.18) | 2.86  (2.07 – 3.77) |
| **HIRI-MSM risk score ≥ 10** | 93.3  (92.3 - 94.2) | 2.05  (0.52 - 3.63) | 2.09  (1.20 - 3.04) |
| **PrEP for all** | 100% | 2.04  (0.66-3.55) | 1.97  (1.16 - 2.86) |

***** The HIRI-MSM risk score was approximated; several modifications were needed to facilitate its calculation with the available iPrEx data (see above).
