# Supplementary material for: Evaluating the impact of policies recommending PrEP to subpopulations of men and transgender women who have sex with men based on demographic and behavioral risk factors
Source: PLoS One. 2019 Sep 19;14(9):e0222183. doi: 10.1371/journal.pone.0222183 (PMC6752862; doi:10.1371/journal.pone.0222183)
Supplement: S3 Table — (DOCX) [file pone.0222183.s004.docx]

**Table S3: Comparison of PrEP recommendations for risk-based PrEP policy and US CDC PrEP guideline.**

|  |  | **Risk-based policy** | |  | |
| --- | --- | --- | --- | --- | --- |
|  |  | *Do not recommend PrEP* | *Recommend PrEP* | | Total |
| **CDC Guideline** | *Do not recommend PrEP* | 279 | 54 | | 333 |
|  | *Recommend PrEP* | 706 | 1403 | | 2109 |
|  | Total | 985 | 1457 | | 2442 |
