# Supplementary material for: Evaluating the impact of policies recommending PrEP to subpopulations of men and transgender women who have sex with men based on demographic and behavioral risk factors
Source: PLoS One. 2019 Sep 19;14(9):e0222183. doi: 10.1371/journal.pone.0222183 (PMC6752862; doi:10.1371/journal.pone.0222183)

**Figure S3: Variable importance summary for Cox proportional hazards regression with lasso models.** Bar charts show how many times a variable was selected across 500 bootstrap datasets.


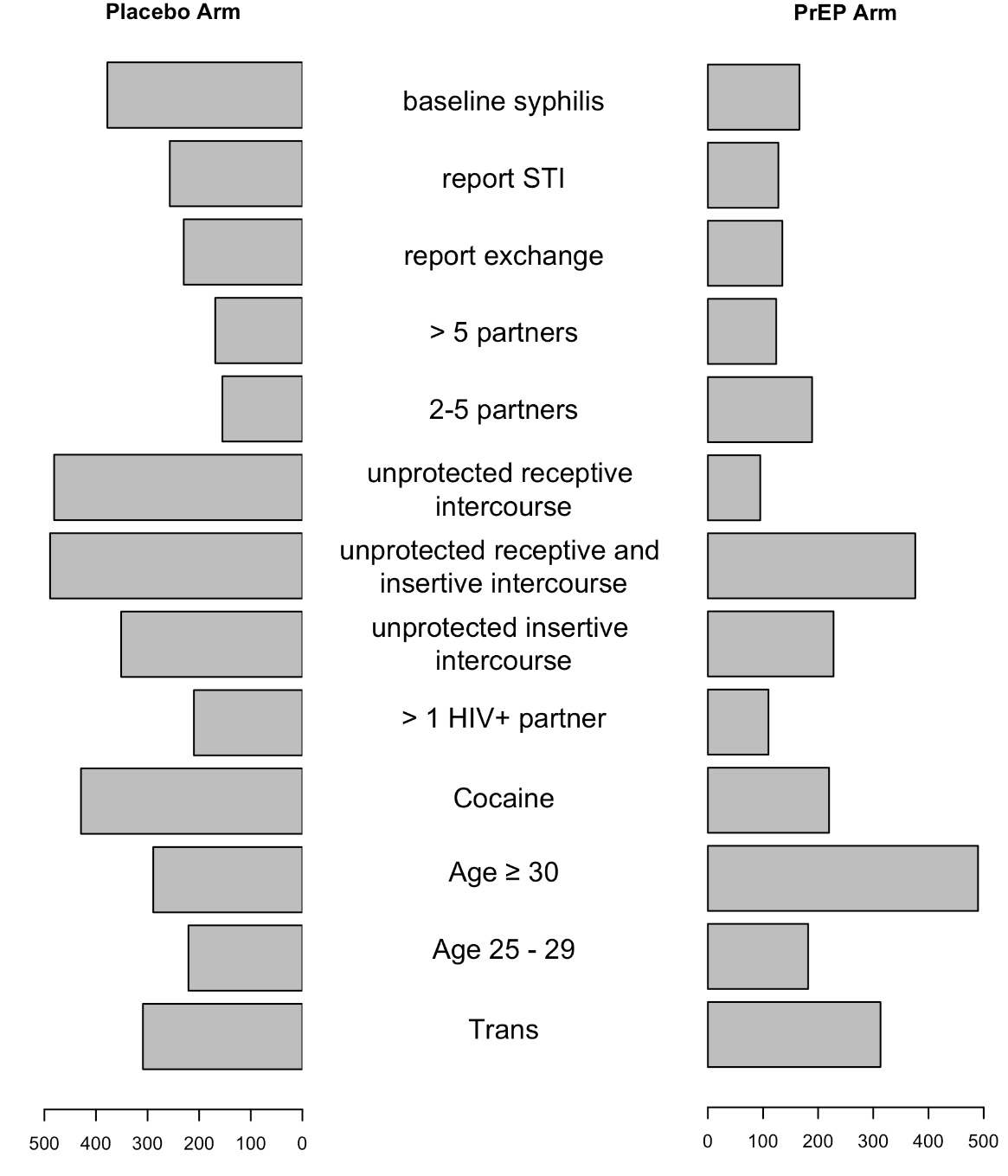

Supplement: S3 Fig — Bar charts show how many times a variable was selected across 500 bootstrap datasets. (DOCX) [file pone.0222183.s007.docx]
