# Supplementary material for: Evaluating the impact of policies recommending PrEP to subpopulations of men and transgender women who have sex with men based on demographic and behavioral risk factors
Source: PLoS One. 2019 Sep 19;14(9):e0222183. doi: 10.1371/journal.pone.0222183 (PMC6752862; doi:10.1371/journal.pone.0222183)

**Figure S4: Flowchart for determining PrEP recommendation for an individual MSM/TGW under the PrEP-benefit-based policy, which is based on a model for PrEP benefit fit to the iPrEx data.** A PrEP benefit threshold of 1.2% is used. Empirical estimates of the size of each subpopulation and of the reduction in 1-year HIV infection risk due to PrEP are shown. The PrEP benefit model describes gradients in PrEP benefit as a function of age, within condomless intercourse subgroups.


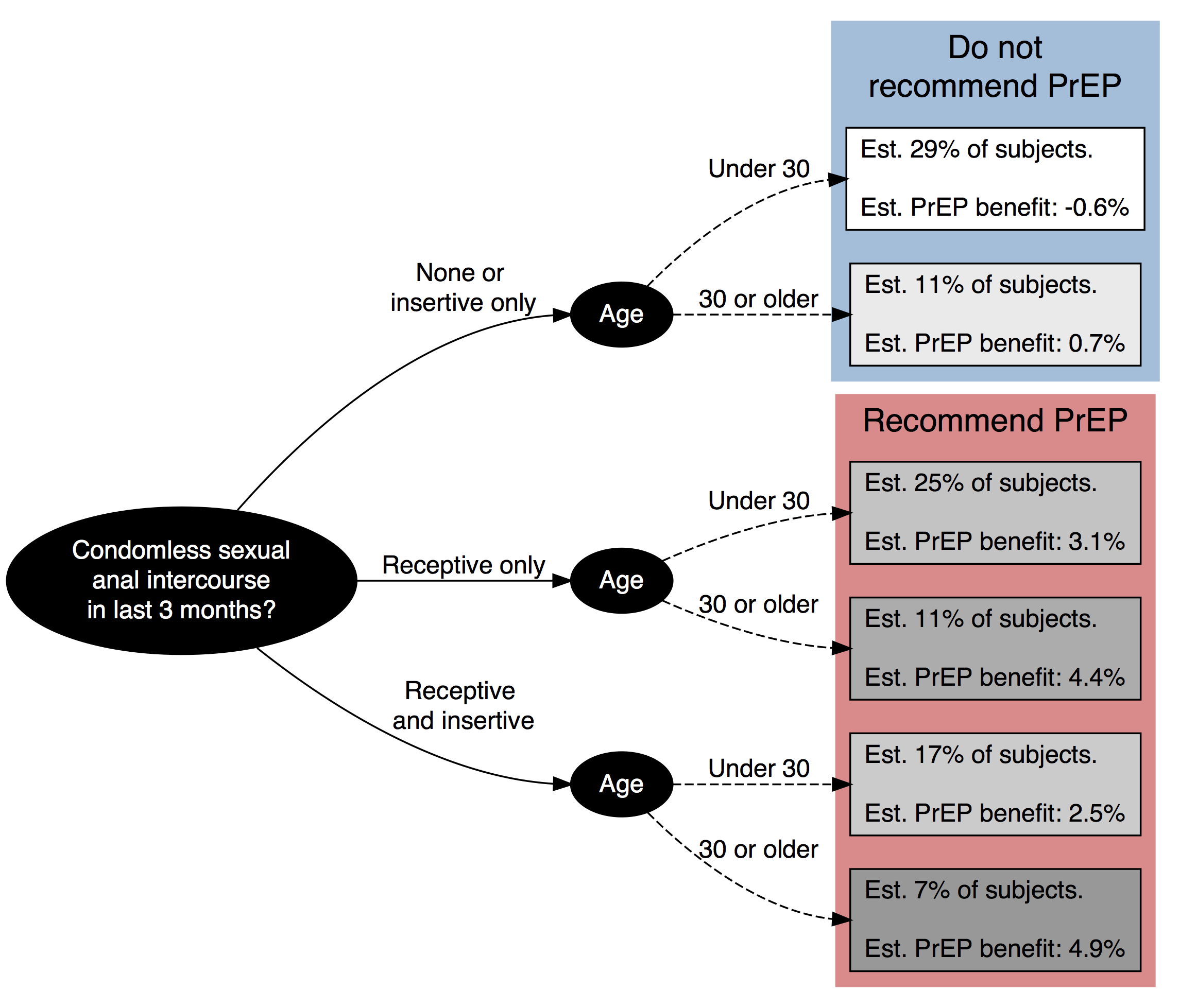

Supplement: S4 Fig — A PrEP benefit threshold of 1.2% is used. Empirical estimates of the size of each subpopulation and of the reduction in 1-year HIV infection risk due to PrEP are shown. The PrEP benefit model describes gradients in PrEP benefit as a function of age, within condomless intercourse subgroups. (DOCX) [file pone.0222183.s008.docx]
