# Supplementary material for: Evaluating the impact of policies recommending PrEP to subpopulations of men and transgender women who have sex with men based on demographic and behavioral risk factors
Source: PLoS One. 2019 Sep 19;14(9):e0222183. doi: 10.1371/journal.pone.0222183 (PMC6752862; doi:10.1371/journal.pone.0222183)

**Figure S5: Distribution of the proportion of times each individual is recommended PrEP across 500 bootstrap datasets, for policies of different types (risk- and PrEP-benefit based policies built using different modelling approaches).** A policy that has more mass near 0 and 1 produces more stable treatment recommendations. PrEP-benefit-based policies use a PrEP benefit threshold of 1.2%.


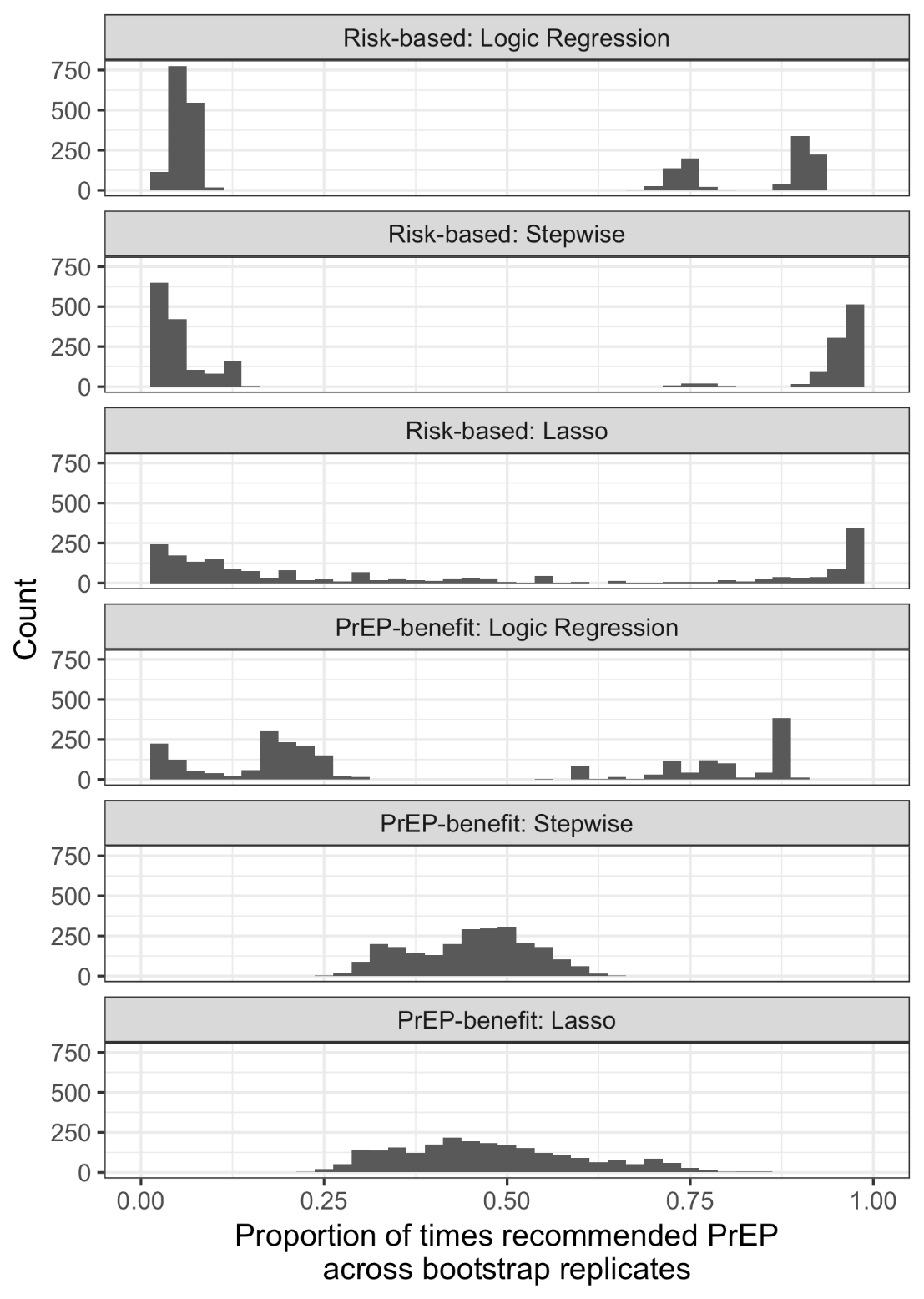

Supplement: S5 Fig — A policy that has more mass near 0 and 1 produces more stable treatment recommendations. PrEP-benefit-based policies use a PrEP benefit threshold of 1.2%. (DOCX) [file pone.0222183.s009.docx]
